# Single-cell analysis technologies for cancer research: from tumor-specific single cell discovery to cancer therapy

**DOI:** 10.3389/fgene.2023.1276959

**Published:** 2023-10-12

**Authors:** Siyuan Chen, Weibo Jiang, Yanhui Du, Manshi Yang, Yihan Pan, Huan Li, Mengying Cui

**Affiliations:** ^1^ Department of Hepatobiliary and Pancreatic Surgery, The Second Hospital of Jilin University, Changchun, China; ^2^ Department of Orthopaedic, The Second Hospital of Jilin University, Changchun, China; ^3^ Department of Orthopaedics, Jilin Province People’s Hospital, Changchun, China

**Keywords:** single-cell sequencing (SCS), circulating tumor cells (CTCs), lineage tracing, tumor heterogeneity, tumor immunotherapy

## Abstract

Single-cell sequencing (SCS) technology is changing our understanding of cellular components, functions, and interactions across organisms, because of its inherent advantage of avoiding noise resulting from genotypic and phenotypic heterogeneity across numerous samples. By directly and individually measuring multiple molecular characteristics of thousands to millions of single cells, SCS technology can characterize multiple cell types and uncover the mechanisms of gene regulatory networks, the dynamics of transcription, and the functional state of proteomic profiling. In this context, we conducted systematic research on SCS techniques, including the fundamental concepts, procedural steps, and applications of scDNA, scRNA, scATAC, scCITE, and scSNARE methods, focusing on the unique clinical advantages of SCS, particularly in cancer therapy. We have explored challenging but critical areas such as circulating tumor cells (CTCs), lineage tracing, tumor heterogeneity, drug resistance, and tumor immunotherapy. Despite challenges in managing and analyzing the large amounts of data that result from SCS, this technique is expected to reveal new horizons in cancer research. This review aims to emphasize the key role of SCS in cancer research and promote the application of single-cell technologies to cancer therapy.

## 1 Introduction

A single cell is the fundamental unit of life activity. It is affected by interactions between genetic mechanisms and the cellular environment to form complex structures such as tissues and organs. The anatomical composition and a description of the interactions, dynamics, and functions at single-cell resolution are essential for a full understanding of the biology of almost all life phenomena, whether normal or diseased (Han et al., 2022).

Continued advances in high-throughput sequencing technologies are giving rise to new genomics, epigenomics, transcriptomics, and proteomics technologies. Single-cell genomics (SCG) can reveal cell genealogy relationships, transcriptomics will aid in replacing the crude concept of marker-based cell types, and epigenomics and proteomics can analyze the functional state of individual cells (Shapiro et al., 2013). The significance of single-cell sequencing (SCS) analysis lies in providing a platform for inferring the cell types and functional status of their ancestors, shedding light on basic underlying questions of biology and medicine. SCS is widely used to study common human diseases, including metabolic, circulatory, chronic, and infectious diseases, as well as clinically challenging neoplasms.

SCS has become an important tool for researchers to explore the gene regulatory networks and cellular dynamics. This review comprehensively analyzed analyzes the prominent experimental results and emerging applications scenarios of SCS in recent years, and outlines the development developing potential of single-cell DNA sequencing (scDNA-seq), single-cell RNA sequencing (scRNA-seq), single-cell assay for transposase accessible chromatin by sequencing (scATAC-seq), single-cell cellular indexing of transcriptomes and epitopes by sequencing (scCITE-seq), single-cell single nucleus chromatin accessibility and mRNA expression sequencing (scSNARE-seq) and data analysis techniques. We summarize the clinical use of SCS in different cell types and the challenges faced in the actual operation process. We also focus on the application of SCS to the detection of circulating tumor cells (CTCs), tumor heterogeneity, drug resistance and immunotherapy, review its status, and speculated on its future development.

## 2 Single cell sequencing technologies

### 2.1 Single-cell DNA sequencing (scDNA-seq)

**FIGURE 1:**
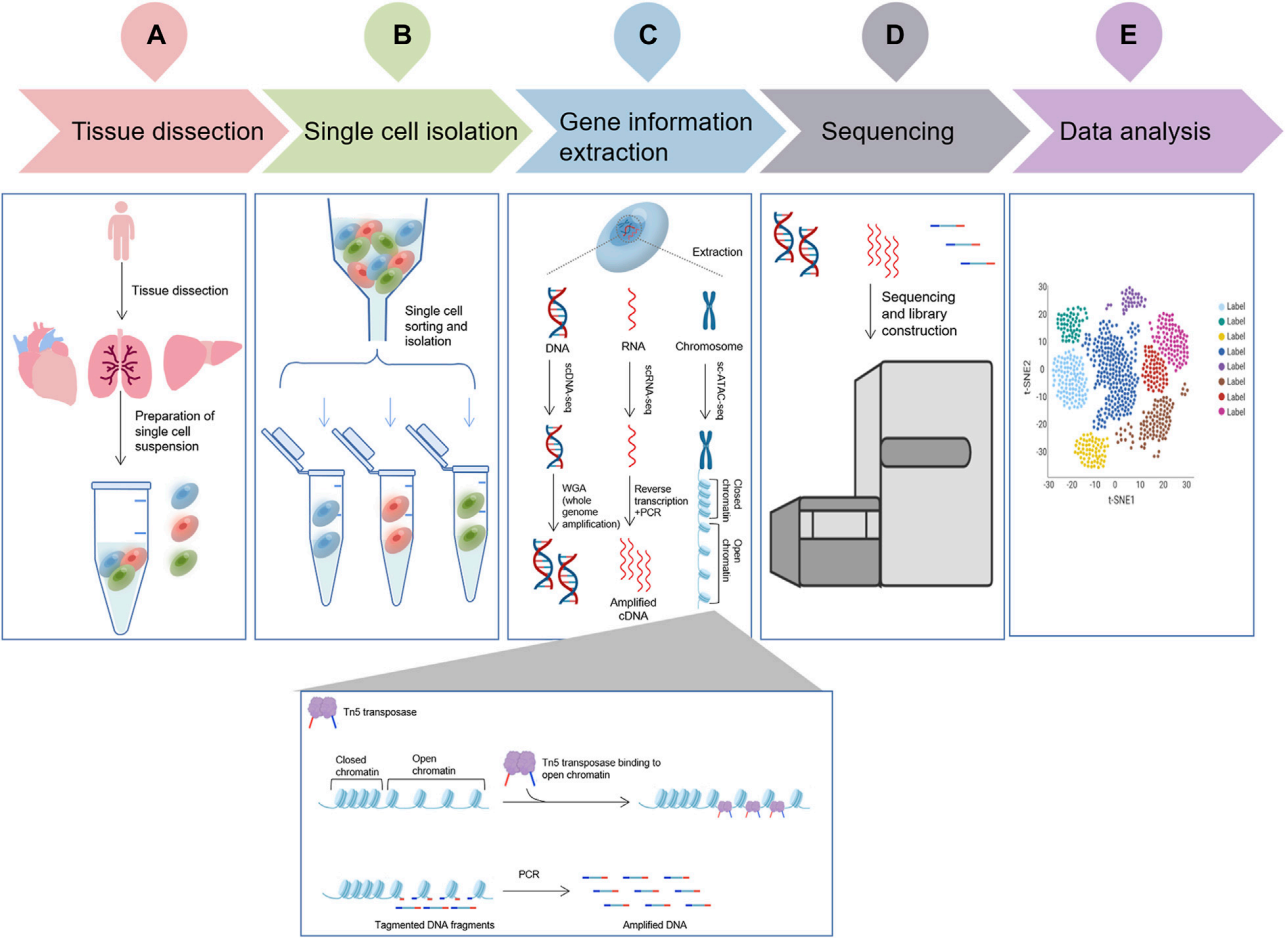
The flowchart of a typical single cell omic sequencing project. **(A)**, Tissue dissection and preparation of single cell suspensions; **(B)**, Single cell isolation (More details about different isolation methods can be seen in Figure 2); **(C)**, Three single-cell technologies (scDNA-seq, scRNA-seq and sc-ATAC-seq) important gene information extraction. ScDNA-seq: DNA is extracted from individual cells using specific kits or techniques, followed by WGA (whole genome amplification) to obtain all DNA sequence information; ScRNA-seq: RNA is extracted from individual cells using specific kits or techniques, RNA is converted to cDNA using reverse transcriptase, and amplification is performed by PCR to obtain all the information of the transcriptome; and sc-ATAC-seq uses the Tn5 transposase, capture chromatin open region, cut nuclear DNA to obtain nuclear DNA fragments, amplified by PCR, obtain all chromatin open region nuclear genome information.; **(D)**, Single cell sequencing by specific platform; **(E)**, Data analysis.

scDNA-seq is a type of DNA high-throughput sequencing technology that can perform massive parallel sequencing of hundreds of thousands to millions of DNA molecules at a time. In scDNA-seq, single cells are isolated and whole genomes of individual cells are amplified, followed by high-throughput sequencing to understand the biological functions of the genes in a single cell (Han et al., 2022) (Figure 1).

**FIGURE 2:**
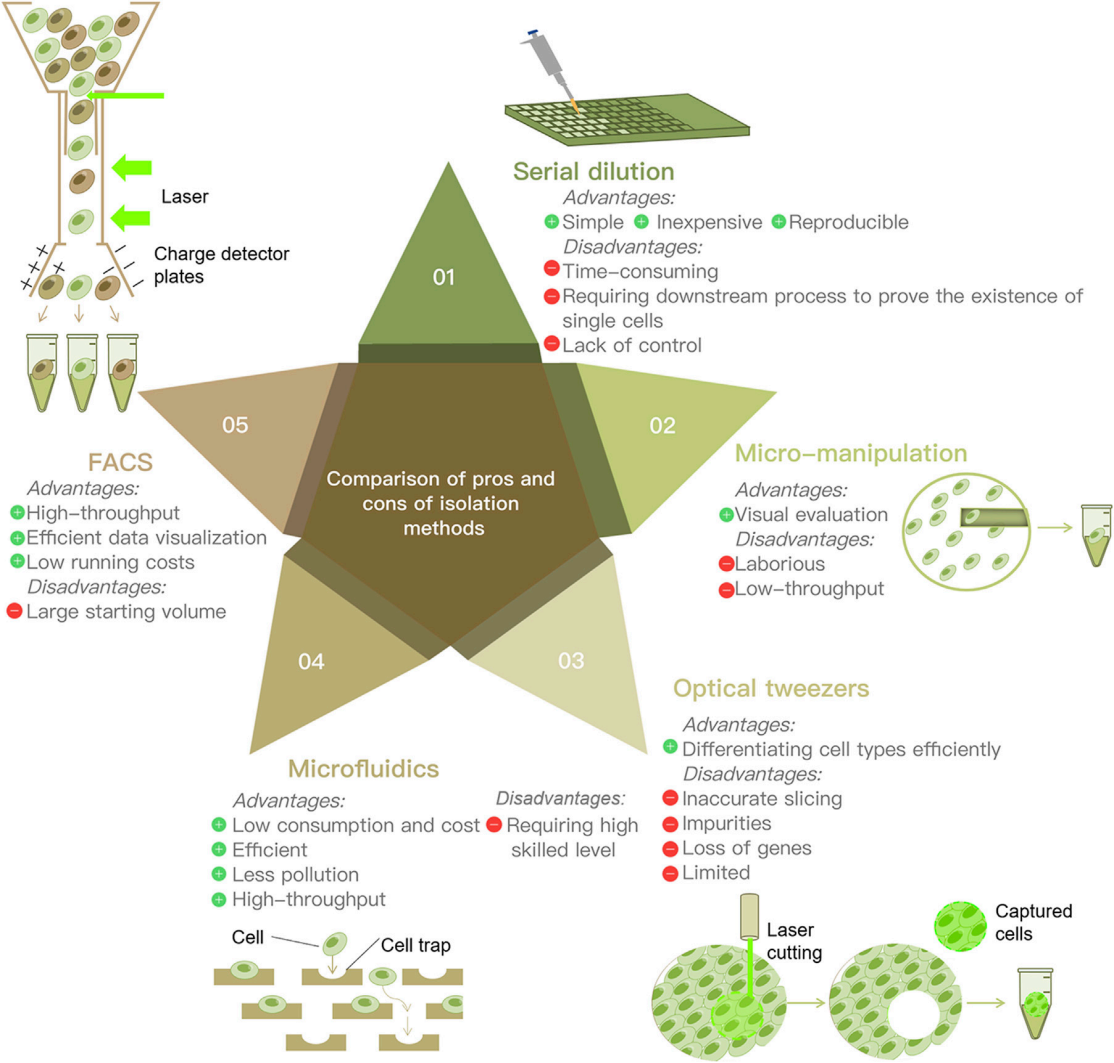
Comparison of the advantages and disadvantages of five main cell separation techniques.

Isolating single cells from environmental samples is the first and the most principal procedure of SCS proceedings. Several single cell isolation methods have been developed, including serial dilution, micro-manipulation, optical tweezers, microfluidic and fluorescence activated cell sorting (FACS). There are various methods for isolating single cells for analysis. Serial dilution is commonly used, because of its simplicity and inexpensiveness (Yilmaz and Singh, 2012); however, it is time-consuming and laborious (Hwang et al., 2018). It is not sufficiently automated and cannot separate a large number of cells at once. Micro-manipulation is a classical method and is suitable for a small number of cells, as well as visual evaluation (Grun and van Oudenaarden, 2015). Nevertheless, the main drawbacks are that it is low throughput and involves the shearing of cells and laborious work. Laser capture dissection, also called optical tweezers, is useful for the isolation of single cells from a complex matrix (Hwang et al., 2018). Combining imaging-based cell selection with optical trapping, it can differentiate cell types without external labeling by biochemical profiling (Saliba et al., 2014). However, inaccurate probe-guided slicing may cause the addition of impurities or the loss of important genes during the dissection progress. Microfluidics has the benefits of low sample consumption, low analysis cost, and precise control (Hwang et al., 2018) (5). Microfluidic devices encapsulate a single cell together with a single bead inside an oil droplet (Chambers et al., 2019). This process provides a sealed environment for isolation, thus reducing the risk of external contamination (Yilmaz and Singh, 2012). The most prominent advantage of microfluidics is that it is high throughput, capturing tens of thousands of cells in a single pass (Chambers et al., 2019). It enables the analysis of rare cell types in a sufficiently heterogeneous biological space (Hwang et al., 2018). FACS is a high-throughput separation method that sorts cells based on a variety of cell characteristics. Random samples of cells can be purified (Grun and van Oudenaarden, 2015) with unrestricted sorting gates. Fluorescently labeled antibodies are used to isolate cells of interest using targeted cell-surface markers (Saliba et al., 2014). Antibodies recognize specific surface markers and enable sorting of distinct populations (Hwang et al., 2018). Thanks to its efficient data visualization and low running costs, FACS has now become the most widely used strategy for single-cell isolation (Saliba et al., 2014) (Figure 2).

Next, to amplify the whole genome or region of interest, multiple displacement amplification (MDA) has become the preferred method for whole genome amplification (WGA) from single cells. Marcy et al. (Marcy et al., 2007) developed a microfluidic device that enables genomic analysis of microorganisms without the need for culture. Dean et al. (Dean et al., 2001) described a simple method for amplifying vector DNA by rolling circle amplification with random primers and phi29 DNA polymerase. Nishikawa et al. (Nishikawa et al., 2015) found that droplets generated by microfluidics show potential as an effective tool for amplification of low-input DNA for SCG, and proposed a new method termed “droplet MDA”, in which isolated genomic fragments can be amplified in these drops without encountering reagent-borne or environmental contaminants.

The analysis of SCS data is difficult. The larger the scale of the experiment and the higher the amount of underlying cells, the greater the burden of data analysis. As the data of SCS continue to increase, scientists are developing new algorithms to address this issue. For example, the new tool SmashCell developed by Harrington et al. (2010) automated genome assembly, gene prediction and annotation of single-cell amplification genomes. Jensen et al. (2009) used the STRING database to calculate single-copy homologous groups and estimate genome integrity. SCS has encountered serious technical challenges for generating complex data output. In a recent article, the researchers outline the available methods, discuss how to carry out experiments and integrate single-cell data (Grun and van Oudenaarden, 2015). The increasing volume of data from SCS studies also requires more powerful computational capabilities. Computational algorithms that can handle large SCS datasets are needed.

scDNA-seq is most widely used in the field of somatic mutation analysis. scDNA-seq analyzes genetic variants and associated driver and concomitant mutations in the stages of tumor initiation, progression, and metastasis, contributing to the classification, prognosis, targeted therapy, and drug resistance analysis of tumor patients.

### 2.2 Single-cell RNA sequencing (scRNA-seq)

The transcriptome is an important tool for studying cellular phenotype and function. The transcriptional process, which involves synthesizing RNA using DNA as a template, represents the initial step in gene expression and plays a key role in the regulation of gene expression. The conversion of cellular RNA molecules into cDNA using non-probe RNA-seq technology, followed by parallel sequencing using next-generation sequencing technology (Metzker, 2010), is increasingly becoming the preferred choice for analysis. However, 1 cell contains only 1–10 pg of RNA. The capture of small amounts of RNA molecules for the preparation of cDNA and the amplification of these cDNA molecules in large quantities are hardly equal or efficient (Figure 1).

In 1990, Norman Iscove’s group accomplished exponential amplification of cDNA molecules using the polymerase chain reaction (PCR), demonstrating for the first time that transcriptome analysis of single cells was feasible. In the early 1990s, Eberwine et al. (1992) invented a new technique to obtain cDNAs from individual living neuronal cells, and then used these cDNAs as templates for transcription to generate RNAs, enabling linear amplification of RNAs. High-throughput RNA sequencing technology (second-generation sequencing) (Weiss et al., 2013) was developed in 2008, and then researchers combined high-throughput sequencing technology with the previously developed nucleic acid amplification technologies to study the single-cell transcriptome in more detail (McCombie et al., 2019). The first single-cell RNA amplification method combined with second-generation sequencing appeared in 2009, namely, Tang’s method (Tang et al., 2009). Tang found that the expression of thousands more genes could be detected by using single-cell transcriptome technology compared to microarray technology (Tang et al., 2009). Since then, many novel techniques have been developed to provide more information with higher repeatability and sensitivity. Quartz-Seq is actually an optimization of Tang’s method that simplifies the experimental process and further reduces amplification by-product generation (Sasagawa et al., 2017). Single-cell Quartz-Seq reveals fluctuations in global gene expression in an individual cell type at the same cell cycle stage. Through barcoding and pooling samples, Cell expression by linear amplification and sequencing (CEL-seq) was developed to overcome the limitation of small starting amounts of RNA with the use of one round of *in vitro* transcription (Hashimshony et al., 2012). Switching mechanism at 5′ end of the RNA transcript sequencing (smart-seq) is a landmark technology developed in 2012 by scientists from the United States and Sweden (Ramskold et al., 2012). Smart-seq and smart-seq2 (Picelli et al., 2014) are based on switching mechanism at 5′ end of RNA template technology (SMART) for amplification and sequencing target RNAs, and there are already more mature commercialized kits that have been widely used.

With the deepening and refinement of applications in scRNA-seq (Zheng et al., 2017), sequencing a few cells no longer meets the requirements of scientific research. It is necessary to simultaneously sequence thousands or even tens of cells at one time to analyze the differences in gene expression between cells. Therefore, there is in urgent need to develop large-scale and low-cost SCS methods. Thus, two teams at Harvard University combined microfluidics with scRNA-seq to develop Drop-seq (Macosko et al., 2015) and inDROP (Klein et al., 2015), respectively. Both technologies utilize a microfluidic device to generate droplets that flow along a very fine channel which encapsulates microbeads with barcodes into the droplets along with the cells, enabling reverse transcription-amplification library building in the droplets while each barcode is attached to a gene in each cell, thus allowing all genes to be determined at once and tracing the cell of origin of each gene. The advent of these technologies has made it possible to analyze the gene activity of thousands of single cells quickly and inexpensively.

A high-throughput single-cell RNA sequencing method was established by a team from the Zhongshan Ophthalmic Center of Sun Yat-sen University through multiple RNA links to analyze the limbal tissue cells of cynomolgus monkeys (Shi et al., 2023). This breakthrough has raised the throughput capability of single-cell full-length RNA sequencing to a new level, while maintaining the sequencing accuracy. These results demonstrate the ability of scRNA-seq to comprehensively analyze the RNA isoforms of different cell types in human tissues, leading to a deeper understanding of the pathogenesis of diseases and potential therapeutic targets, and providing new directions for novel drug development.

### 2.3 Single-cell assay for transposase accessible chromatin by sequencing (scATAC-seq)

Single-cell assay for transposase accessible chromatin sequencing (scATAC-seq) is a tool for simultaneous profiling of accessible chromatin and protein levels, based on 10× Genomics Chromium™ dynamic microfluidic technology and using Tn5 transposase to cut open chromatin and form short fragments (Mimitou et al., 2021). The accessibility of chromatin is closely associated with transcriptional regulation (Buenrostro et al., 2015). Now, scATAC-seq is gradually entering the field of single-cell omics research with the advancement of the technology and its application value (Figure 1).

Innate lymphoid cells (ILCs) have been found to receive signals from other cells in the tissue microenvironment and play a vital immune role on the mucosal surface. Bielecki et al. (2021) confirmed the potential of skin ILCs to switch between natural, resting, and ILC2 effector states after disease induction through *in vitro* experiments, scATAC-seq, and *in vivo* fate mapping, contributing to a better understanding of the pathological process of skin inflammation in psoriasis caused by ILC responses to different signals that are generated. scATAC-seq analysis can be also used to draw the single-cell epigenome and a transcriptional map of influenza vaccine immunization (Wimmers et al., 2021).

scATAC-seq can explore homeopathic regulatory sequences and gene expression regulation by detecting the openness of chromatin. Combining with single cell transcriptome sequencing, it can detect the gene regulatory network (Buenrostro et al., 2015; Cusanovich et al., 2015), potential chromatin structure and key transcription factors of each cell type (Cusanovich et al., 2018). For example, scATAC-seq combined with scRNA-seq analysis is used to study the regulation of hematopoiesis during human development (Buenrostro et al., 2018), explore heterogeneity of breast cancer (Pervolarakis et al., 2020), investigate the underlying gene regulatory networks and epigenetic changes that drive cell fate transitions during early heart development (Jia et al., 2018).

In summary, scATAC-seq technology is becoming increasingly prevalent in the study of tumors (Pervolarakis et al., 2020), embryonic development (Wu et al., 2018), and immune diseases (Satpathy et al., 2019). This technology will provide scientists with a robust technical tool for understanding the dynamic changes in chromatin during different life processes, the downstream regulation of gene transcription and other related aspects.

### 2.4 Single-cell cellular indexing of transcriptomes and epitopes by sequencing (scCITE-seq)

High-throughput scRNA-seq has revolutionized our comprehension of intricate cell populations, however, it falls short in offering phenotypic insights such as cellular surface protein expression. scCITE-seq is a method that combines highly multiplexed protein marker detection with unbiased transcriptome profiling for thousands of single cells (Stoeckius et al., 2017). It enables simultaneous access to cellular gene expression data and surface protein marker information, thereby affording a more comprehensive description of cellular phenotypic properties.

Unlike traditional scRNA-seq, scCITE-seq requires surface protein labeling of cells before processing. Afterwards, the cDNA library and “surface protein” library formed by mRNA reverse transcription were constructed through PCR amplification. The two constructed libraries can be subjected to high-throughput sequencing for data analysis. scCITE-seq data includes protein and transcriptome information, which can link the immune phenotype of cells with gene expression, and annotate cell subpopulations or rare cell types more accurately.

By establishing a mutant mouse, Nathan Salomonis’ team (Muench et al., 2020) combined scCITE-seq and scATAC-seq techniques to analyze the pathogenic mechanism of severe congenital neutropenia in children caused by GFI1 mutation. The author further explored the surface protein expression levels in various cell subpopulations from the sequencing results of scCITE-seq, and validated the chromatin defects and a series of molecular dysfunctions caused by GFI1 mutations. In the future, the utilization of scCITE-seq analysis on tumor specimens is expected to detect tumor cells and various infiltrating immune cell populations in the tumor microenvironment (Stoeckius et al., 2017).

scCITE-seq is believed to be highly advantageous in the profound characterization of tumor heterogeneity and the development of novel therapeutic modalities. Wu et al. (2021) introduced a single-cell method known as SCS subtype to identify innate subtypes, thus shedding light on the heterogeneity of recurrent tumor cells in breast cancer. The immunophenotypic analysis by scCITE-seq produces high-resolution immunoprofiles, including the identification of novel PD-L1/PD-L2+ macrophage populations associated with clinical outcomes. The spatial arrangement of the stromal immune microenvironment within tumors offers valuable insights into anti-tumor immune regulation (Stoeckius et al., 2017).

### 2.5 Single-cell single nucleus chromatin accessibility and mRNA expression sequencing (scSNARE-seq)

scSNARE-seq refers to single nucleus chromatin accessibility and mRNA expression sequencing (Chen et al., 2019). The main feature of scSNARE-seq is the ability to perform large-scale SCS combining nuclear chromatin accessibility and mRNA expression.

The fundamental approach involves the use of Tn5 transposase to capture chromatin accessibility within the permeable nucleus before droplet formation. This facilitates the co-packaging of accessible genomic regions and mRNA from individual nuclei within the same droplet. Simultaneously, a “sandwich oligonucleotide” has been devised, which complements the linker sequence introduced at the 5′ end of the transposon and concludes with poly-A, allowing for its capture by beads carrying poly-T. Subsequently, the encapsulated mRNA and fragmented genomic DNA are liberated by heating the droplets, and a library is constructed for subsequent sequencing.

Chen et al. (2019) also mapped brain samples of newborn and adult mice by scSNARE-seq. By cluster analysis, they delineated at least 20 distinct cell types within a pool of 16,000 brain cells. Notably, scSNARE-seq, in contrast to the existing scATAC-seq method, concurrently integrates transcriptomic data from each cell, enabling a more precise cell type identification. The data generated by SNARE-seq exhibit enhanced quality and illuminate tissue complexity by analyzing the input and output of transcriptional regulatory units. The approach offers comprehensive and distinctive sequencing advantages, including heightened sensitivity, specificity, resolution, increased measurement throughput, and ease of implementation. These properties have a profound significance in creating single-cell maps for various human organ systems and advancing clinical research on diseases.

### 2.6 Translational of single cell technologies to cancer studies

SCS analysis distinguishes between functionally healthy cells and cancer cells at different stages of tumor development. The application of SCS in large-scale cancer research can be categorized into three key areas. First, perform comprehensive omics-level SCS on tumor tissues with the selection of appropriate omics sequencing methods according to the research objectives (Zhou et al., 2020). Next, analyze the constituents of cells based on omics sequencing data (Gong et al., 2021). For tumor cells, we should pay attention to heterogeneity, while for immune cells, we should focus on cell subtypes and states, leading to the establishment of tumor-related indicators or characteristics (Zhou et al., 2021). Finally, select a suitable dataset with tumor therapy strategies for further clinical validation in terms of treatment selection, treatment monitoring and response, survival prognosis, and other aspects. By adopting the multifaceted approach, SCS technology enhances our ability to decipher the complexities of cancer biology and provides valuable insights for more effective cancer diagnosis and treatment.

## 3 Data analysis technologies

SCS data constitutes a high-dimensional and intricate dataset. To efficiently process and analyze single-cell sequencing data, particularly for cell subtype identification, it is often necessary to commence with dimensionality reduction (Malepathirana et al., 2022). Dimensionality reduction aims to optimize high-dimensional data by preserving critical features from the original dataset and projecting them into a lower-dimensional space, thereby facilitating data representation in a two-dimensional or three-dimensional format. Common dimensionality reduction methods include principal component analysis (PCA) (Jolliffe and Cadima, 2016; Ben Salem and Ben Abdelaziz, 2021), *t*-distributed stochastic neighbor embedding (*t*-SNE) (Kobak and Berens, 2019), uniform manifest approximation and projection (UMAP) (Becht et al., 2018), and scvis (Ding et al., 2018). PCA is a linear dimensionality reduction method that converts high-dimensional data into low-dimensional representations through linear transformation, preserving the maximum variance. While t-SNE is a nonlinear dimensionality reduction method that maps high-dimensional data to low-dimensional space by optimizing the similarity between samples, preserving the local structure between samples (Zhou and Sharpee, 2022).

Clustering aims to group similar cell categories together (Gao, 2018). In the dimensionality reduction clustering map of SCS analysis, the gene expression of each cell is displayed on a two-dimensional plane, and cells that share similar gene expression patterns are clustered together. For each identified cell cluster, we can pinpoint the cluster-specific marker genes expressed within it using differential gene analysis, aiding subsequent cell type annotation. Typically, differential genes between a cluster and all other clusters are used as markers for that cluster. After identifying cell clusters and their associated marker genes, the next step is to determine the cell types of these clusters, a process known as cell type annotation. A cell cluster can be classified as the corresponding cell type if its marker genes coincide with those of a cell type. This annotation step is important in single-cell analysis, and various cell automated annotation tools such as single recognition or single-cell cluster-based annotation toolkit for cellular heterogeneity (scCATCH), which can aid in cell type assignment (Brent, 2008). To enhance annotation accuracy, single-cell public databases (e.g., CellMarker, PangLaoDB, CancerSCEM, SingleCellPortal, etc.) can be consulted to access reference datasets or known cell type markers, allowing for improvements in annotation quality.

To accurately elucidate the functional bias and biological significance of specific cell populations, it becomes crucial to perform functional enrichment analysis on the target set of differentially expressed genes, which is known as differential expression analysis (Anders and Huber, 2010). Several other important aspects deserve further attention and exploration in the field of SCS analysis. These include the synergistic application of scRNA-seq and clustered regularly interspaced short palindromic repeats (CRISPR) screening (Ji et al., 2020), as well as the comprehensive analysis of scRNA-seq data alongside multi-omics approaches, encompassing single-cell methylation and transcriptome sequencing (scMT-seq) (Hu et al., 2016) and spatial transcriptomics. In the field of SCS research, analytical algorithms and tools still possess substantial potential to enhance data exploration and our understanding of cell functionality.

## 4 Applications of SCS

### 4.1 Cell-type-specific SCS

Intercellular variability and heterogeneity are essential and intrinsic features of cell populations. Even a “pure” cell type will have heterogeneous gene expression from the same genetic structure. In addition, gene expression may change during the cell cycle and can be affected by randomness within the gene expression system and external microenvironments (Junker and van Oudenaarden, 2014). However, when large numbers of cells are used for genome-wide analysis, the apparent heterogeneity between cells masks the heterogeneity within a “pure” cell. SCS technologies emerged as a result. The obvious advantage is that these technologies allow the profiling of single-cell heterogeneity in a comprehensive and unbiased way without prior whole-cell population sequencing (Wen and Tang, 2016). The application of these methods in different types of cells has led to exciting new discoveries. More details and the significance of SCS in diverse cell types are discussed in this section.

#### 4.1.1 Stem cells

The application of SCS in stem cells can help reconstruct the core gene regulatory network within each cell during differentiation and establish causal relationships between genotype and phenotype, while also providing a comprehensive understanding of the complexity of gene regulatory networks under physiological and pathological conditions, offering new insights into the biological basis of human development and disease (Wen and Tang, 2016).

##### 4.1.1.1 Small intestinal stem cells

A research team led by Alex and Wrana (Ayyaz et al., 2019) has identified a unique class of stem cells which activated after injury to the small intestine to maintain homeostasis of the stem cell pool and promote regeneration of the small intestinal epithelium. scRNA-seq made it possible to analyze the regenerating mouse intestine and identify a unique, injury-induced quiescent cell type which termed regenerative stem cells (revSCs).

##### 4.1.1.2 Hematopoietic stem cells

Dong et al. (2020) tracked the differentiation process of transplanted hematopoietic stem cells in the host using scRNA-seq. Through tracking the dynamics, the absence of significant hematopoietic stem cell expansion during the first week post-transplantation was found, revealing that hematopoietic stem cells transplanted in myeloablative recipients at an early stage were not available for kinetics and fate selection.

##### 4.1.1.3 Embryonic stem cells

scRNA-seq analysis of human preimplantation embryos and human embryonic stem cells (hESCs) constructs an integrated framework for the transcriptome view. Klein et al. (2015) revealed the population structure and differentiation heterogeneity without leukemia inhibitory factor (LIF) by performing scRNA-seq on mouse embryonic stem cells (ESCs). Budnik’s team also did similar study to quantify the heterogeneity and dynamic changes in protein expression at the single cell level in mouse ESCs during differentiation (Budnik et al., 2018). The difference between two studies is the former applies scRNA-seq while the latter applies single cell proteome analysis. The second study defined cell types and inferred potential relationships between cell types and specific protein abundance. Comparative analysis between single-cell proteomes and transcriptomes has shown that covariation exists between mRNA and protein levels, and that many genes can play a synergistic regulatory role at both mRNA and protein levels.

#### 4.1.2 Primary and metastatic tumor cells

The detection of oncogenes and tumor suppressor genes in tumor cells by SCS can determine whether a tumor has metastasized, providing important perspectives on the differentiation and diagnosis of primary and metastatic tumors and helping to determine different treatment strategies (Ohgami et al., 2015). In addition, scRNA-seq profiles of individual cancer patients have shown that intercellular adhesion molecules, tumor neovascularization, and extracellular matrix adhesion and degradation (Eble and Niland, 2019) are closely related to tumor metastasis.

There are significant differences between primary and metastatic tumors in terms of their tumor heterogeneity, drug resistance, and tumor microenvironment. Components of the tumor microenvironment, such as tumor-associated macrophages (TAM), play an important role in promoting tumor metastasis (Fu et al., 2020). SCS technology can be used to examine the relationships between tumor metastasis and tumor heterogeneity, tumor drug resistance, and the tumor microenvironment, thus helping to differentiate and diagnose primary and metastatic tumors and to suggest new treatment strategies accordingly. A colorectal cancer study (Okamoto et al., 2021) used SCS to determine the cell composition of tumors in patients with primary and metastatic colorectal cancer, indicating the cellular heterogeneity of metastatic tumors and primary tumors. Additionally, SCS has been used in breast cancer for the study of transcriptional heterogeneity in primary and metastatic tumors, providing a therapeutic target for preventing the metastasis and spread of breast cancer (Davis et al., 2020). SCS can accurately detect heterogeneous dynamic changes among tumor cells at different times and in different spatial locations. Pan et al. (2020) used SCS to analyze cancer stem cells of a pair of primary and metastatic collecting duct renal cell carcinomas, and found that cancer stem cells can transform into primary and metastatic collecting duct renal cells in a spatiotemporal manner.

In conclusion, the results above suggest that SCS can identify potentially critical factors in tumorigenesis and metastasis at the single-cell level to guide the development of precise treatment regimens and to track the lineage of metastatic cells.

#### 4.1.3 Circulating tumor cells (CTCs)

Circulating tumor cells (CTCs) refer to tumor cells that can penetrate the basement membrane, invade the surrounding tissues and enter the peripheral circulating blood (Paoletti and Hayes, 2016). They are then transmitted to distal tissues, and exude, adapt to the new microenvironment, finally “seed”, proliferate, and form metastatic tumor. Therefore, early detection of CTCs in blood is an important guide for prognosis, efficacy evaluation and individualized treatment of patients (Zhu et al., 2018). Circulating tumor DNA (ctDNA) is a cell-free extracellular DNA found in body fluids such as blood, synovial fluid and cerebrospinal fluid (Lin et al., 2021). It is mainly composed of single- or double-stranded DNA or mixtures of both, and exists either as a DNA-protein complex or as free DNA. ctDNA is derived from shedding tumor cells or apoptotic cells which are released into the circulatory system. Chae and Oh (2019) showed that ctDNA can be used to detect the presence of microscopic residual disease (MRD) after surgical resection of several cancers, and that MRD will help to identify patients at risk of recurrence and thus guide treatment decisions for resettable cancers.

**FIGURE 3:**
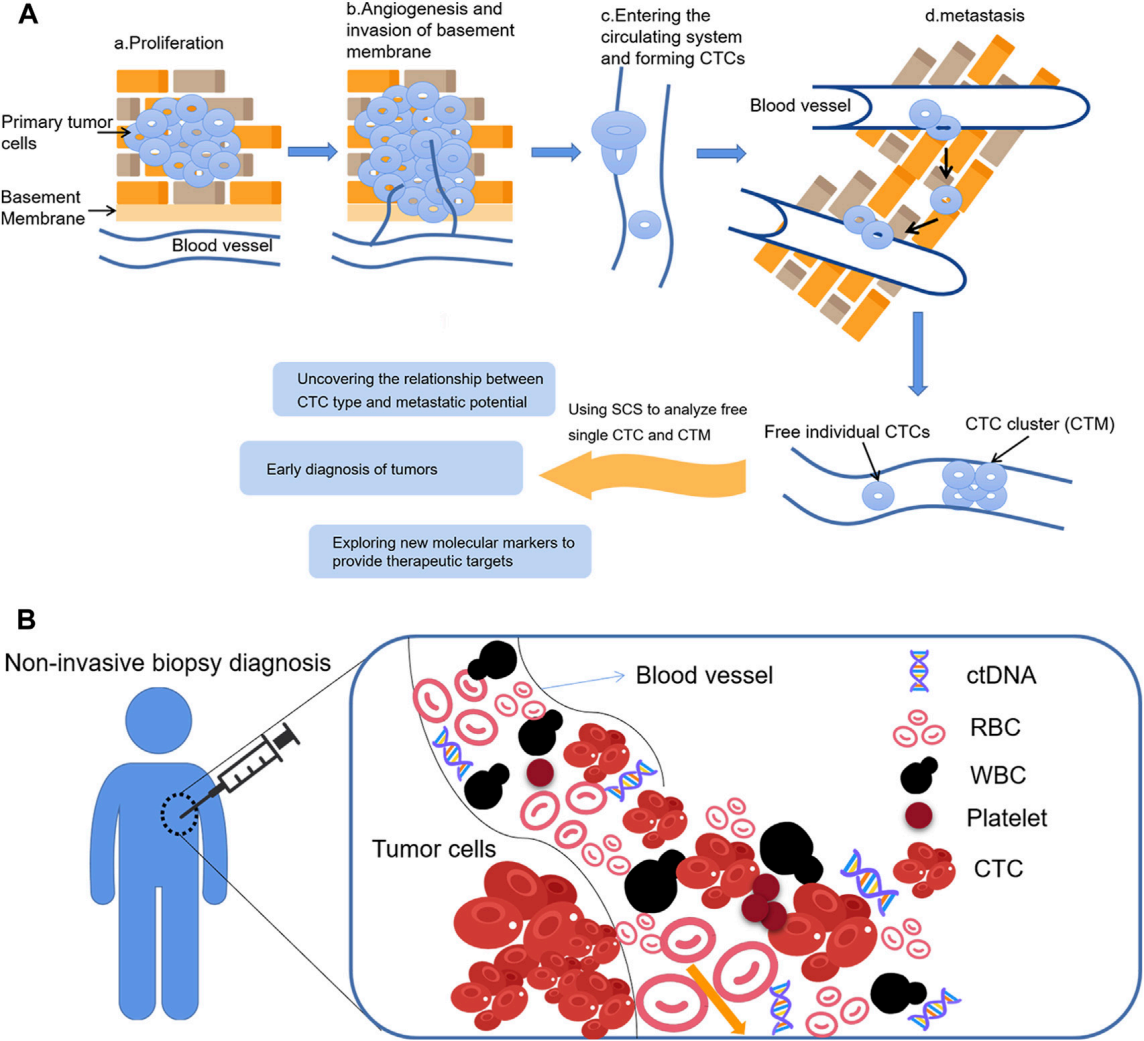
Application of SCS in CTCs **(A)** The formation of CTCs and CTCs analysis; **(B)** Circulating tumor cells as a material for liquid biopsy.

CTCs are present in peripheral blood in different forms, both as free individual CTCs and as aggregated cell clusters (Amintas et al., 2020), known as circulating tumor microemboli (CTM). Tumor cells undergo epithelial mesenchymal transition (EMT) (Jie et al., 2022) during their entry into peripheral circulation, resulting in different types of CTC, including those with an epithelial phenotype, those with a mesenchymal phenotype, and those with a mixed epithelial and mesenchymal phenotype. During EMT, the expression of epithelial markers such as E-cadherin, EpCAM, and cytokeratins decreases, while the expression of mesenchymal markers such as N-cadherin, vimentin, and fibronectin increases. CTM and mesenchymal phenotype CTCs have a higher metastatic potential (Umer et al., 2018), and CTC testing may help to monitor tumor dynamics and assess the treatment efficacy and risk of recurrence in real time by capturing the CTCs in peripheral blood and monitoring trends in the CTC type and number, also known as “liquid biopsy” (Figure 3). Findings in breast and colon cancers have attached great importance to the discovery of markers that can capture CTCs independently of their EMT status. Vimentin may be a suitable candidate biomarker to detect and isolate CTCs.

Liquid biopsy targeting CTCs is one of the most important breakthroughs in precision medicine. The presence or absence of CTCs in the patient’s blood, and their quantity, represent both the ability of the primary tumor to infiltrate into the blood vessels and the possibility of metastases forming in distant organs. Therefore, detecting and counting the number of CTCs can indicate the degree of malignancy of the tumor and the risk of metastasis, which has important diagnostic and prognostic value.

Wankhede et al. (2022) evaluated the overall survival and disease-free survival of CTC-positive and negative patients with early-stage non-small cell lung cancer (NSCLC) respectively by meta-analysis, and showed that CTC-positive patients have a higher risk of recurrence, which can help to guide treatment by stratifying the survival outcome of early-stage NSCLC patients in future clinical applications. He et al. (2017) used the New CellCollector to capture CTCs from blood in patients with lung nodules, healthy volunteers and lung cancer, then used low-dose computed tomography (LDCT) combined with CTC analysis to screen for lung cancer, and performed NGS analysis through WGA. The results showed that CTC were positive in the lung cancer group, while no “CTC-like” events were detected in the healthy group. This suggests that CTC combined with high-throughput sequencing will be a promising method to screen for early-stage lung cancer and has great application prospects in precision medicine. CTC analysis with SCS may contribute to understand the mechanism of mutation in tumor metastasis. Lohr et al. (2014) performed SCS of CTCs, primary tumors, and metastatic lymph nodes from prostate cancer patients, and detected CTC exon mutations in early-stage and stem-cell mutated tumor samples. As mentioned previously, CTC clusters, consisting of aggregated CTCs, have a greater possibility of metastasis compared with individual CTCs (Umer et al., 2018), and metastasis is an important biological feature of advanced malignant tumors. This was further confirmed by Pineiro et al. (2020), who studied the role and metastatic potential of CTC clusters using breast cancer as an example. Additionally, Herath et al. (2020) emphasized the importance of comprehensive characterization of CTC clusters, as heterotypic clusters can provide a mechanism for immune evasion. This study provides a detailed understanding of the cellular interactions within clusters and helps to guide the development of approaches to reduce CTC cluster metastasis. CTCs can reveal genetic information about the tumor origin more accurately than information obtained from individual diagnostic biopsies. Pancreatic cancer is known as the “king of cancers” because of its high malignancy, late onset, and a 5-year survival rate of less than 5%. There is no clear tumor marker for screening and detection of early cases, but CTCs are an emerging biomarker that could assist the early diagnosis and staging of pancreatic cancer (DiPardo et al., 2018).

However, CTCs are a heterogeneous population that may differ from one another in terms of various factors, including cell size and morphology, molecular phenotype, degree of activity, metastatic potential, and proliferative potential. CTCs may exist as single cells or may aggregate with one another or with blood-derived cells to form tiny multicellular aggregates. SCS of CTCs avoids the interference of tumor heterogeneity by comparing the differences between the single-cell genomes, transcriptomes, and the epigenetic group of the primary and metastatic tumors, providing a new dimension to the understanding of cancer biology. CTC analysis can also explore more detailed information at the genetic level, further explore the tumor microenvironment and immune escape-related mechanisms, and guide new directions in anti-tumor drug development (Xu et al., 2021).

Reza et al. (2021) used a simple microfluidic device to screen individual CTCs in a dynamic state to obtain an accurate atlas of the heterogeneous expression of various protein biomarkers in therapy. This automated assay can help to monitor the malignant biological behavior of tumors and provides an important window into understanding tumor heterogeneity. Thiele et al. (2019) use a single-cell proteogenomic approach combined with a high-definition SCS workflow to investigate CTC heterogeneity and understand their full potential in the study of tumors. Single-cell proteomic analysis of CTCs provides critical protein-level data, which can give key information such as the malignancy of the tumor tissue and the activation status of signaling pathways, allowing for more precise treatment planning. Miyamoto and colleagues (Miyamoto et al., 2015) identified the expression of androgen receptor (*AR*) gene mutations and splice variants in circulating prostate tumor cells by scRNA-seq, further demonstrating that single-cell analysis of CTC can reveal tumor genetic heterogeneity, and that this heterogeneity is a cause of treatment failure. However, most current CTC analyses are based on CTC epithelial biomarkers, which may not be expressed in some tumor types. They have not been analyzed at the genomic level, so individual CTC profiles or patient genomic profiles are lacking. Lim et al. (2019) investigated the feasibility of analyzing longitudinal samples for mechanisms associated with acquired therapeutic resistance by genomic analysis of the whole genome/exome of CTCs, performing WGA and quality control of the amplified DNA products prior to sequencing. Thus, SCS is predicted to allow the development of personalized tumor treatment plans for cancer patients in future.

In conclusion, the specific application scenarios of CTC testing in clinical settings include early screening of high-risk groups, accurate staging of confirmed patients, post-operative monitoring of recurrence and metastasis in early-stage patients, prognosis determination before the start of treatment and efficacy evaluation after each cycle of treatment in advanced patients, and real-time analysis of molecular targets to predict the efficacy of relevant drugs, providing important assistance for the whole process of patient management. We should be cautiously optimistic about the clinical application of CTCs, and it is expected that this will help medical treatment in the field of oncology to take radical steps in the future.

### 4.2 Lineage tracing

The combination of scRNA-seq and cell lineage tracing analysis provides a better understanding of the origin of cell populations and their biological functions, leading to the development of more targeted and personalized drugs. Wagner and Klein (2020) used SCS methods to comprehensively document the alterations of cell states and lineage information during organ development, providing insight into the molecular mechanisms involved in genealogical development. This may be helpful to provide clues to explore the origins of cancer. Kumar et al. (2022) established a single-cell profiles of the genealogical status of gastric cancer by orthogonal validation of spatial transcriptomics, independent bulk RNA-seq cohorts. They identified 34 different cell lineage states, including new rare cell populations. Many of the genealogical states showed distinct cancer-related expression profiles, and the results of this study provide a high-resolution molecular resource for genealogical states within and between patients with different gastric cancer (GC) subtypes. Once a cell lineage has been established, understanding the structure and function of a cell at the single-cell level can be used to trace the lineage of cells from which it came, providing important information about its ancestral cells.

The application of scRNA-seq offers the possibility to understand the fundamental mechanisms of organism evolution trajectory and help investigate the genesis of diseases. Paik et al. (2020) showed the full implications of single-cell transcriptome sequencing using cardiovascular cells as an example: detecting rare cell populations, constructing lineage trajectories, identifying intercellular interactions. Fu et al. (2019) compared the gene expression in an individual kidney glomerular cells of diabetic and normal mouse using scRNA-seq. Dynamic changes in the pattern of expressed genes showing that vital factors underlying the pathophysiology of diabetic kidney disease progression and provide potential new therapeutic approaches. Kim et al. (2020) featured 208,506 cells based on the single-cell transcriptome profile of metastatic lung adenocarcinoma and identified subtypes of cancer cell that deviated from the normal differentiation trajectory and dominated the metastatic stage.

### 4.3 Tumor heterogeneity and drug resistance

**FIGURE 4:**
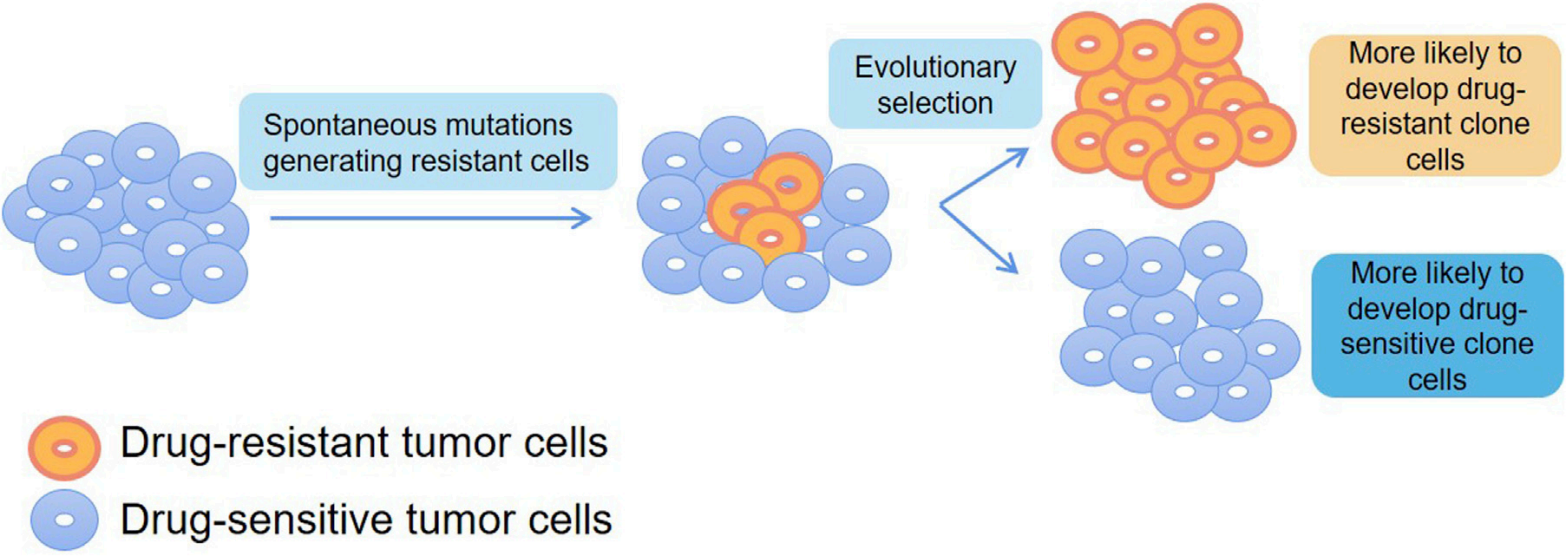
Application of SCS in drug resistance.

Frede et al. (2021) found that different transcriptional states coexist in individual cancer cells and the use of differential transcriptional regulation and enhancer reassociation are the basis of these alternative transcriptional states by using scRNA-seq and scATAC-seq in multiple myeloma. More importantly, the treatment generated a unique immunotherapeutic target, such as CXCR4, that could be used to overcome treatment resistance. Thus, this study using SCS as an analytical tool depicts how cellular plasticity can be translated into drug-resistant immuno-oncology treatment opportunities. Chambers et al. (2019) studied non-malignant lung diseases with aberrant cell differentiation and heterogeneous microenvironment by combining heterogeneity analysis and cell lineage tracing with scRNA-seq. Accurate resolution of clones, which has important implications for providing information about changes in gene expression and insight into the peritumor microenvironment and inflammatory cellular environment, and may inform drug sensitivity or resistance. Under the pressure of chemotherapeutic agents and targeted drugs, spontaneous mutations cause a fraction of tumor cells to acquire drug resistance. Therefore, proteomic analysis of tumor cells at the single-cell level can more accurately establish the relationship between gene mutations and protein expression and thus infer different mechanisms of drug resistance, providing theoretical support for subsequent drug dosing and the development of novel anticancer drugs (Figure 4).

The future drug screening models will certainly tend to observe the efficacy and toxicity of drug candidates on different types of cells with different functions, and the most ideal cell source should be human primary cells. Single-cell proteomics analysis breaks the bottleneck of sample size and is well suited for high-throughput drug screening on primary cells to clarify the mechanism of drug action and potential toxicity, and to improve the success rate of drug development (Kelly, 2020).

### 4.4 Tumor immunotherapy

Immune cells are highly differentiated in cell type and function and maintain immune homeostasis *in vivo* by finely regulating each other. Conventional sequencing methods mask the characteristics of individual cells, so SCS analysis of a single type of immune cell is essential to reveal the mechanisms of immune-related diseases and to contribute to the development of more effective individualized treatment protocols to guide clinical care and predict prognosis (Liu et al., 2022).

Lin’s team (Yang et al., 2023) from the Chinese Academy of Medical Sciences used scRNA-seq to collect the functional role of apolipoprotein B mRNA editing enzyme catalytic polypeptide (APOBEC) mutagenesis in 169 patients with esophageal squamous cell carcinoma (ESCC), and evaluated the characteristics of cancer cell immune infiltration and found that APOBEC mutagenesis can prolong the overall survival of ESCC patients, which may show greast potential in prognostic value for immunotherapy.

The collection of sequence diversity is called the immune repertoire (IR), and single-cell immune repertoire sequencing (scIR-seq) has recently been developed. Using high-throughput sequencing, a large number of single-cell gene expression profiles and immunomic library data can be obtained in parallel, enabling simultaneous high-throughput sequencing of gene transcripts and immunomic libraries at a single-cell resolution (Tian et al., 2022). Zheng et al. (2021) Zheng et al. (2021) systematically analyzed the heterogeneity and dynamics of tumor-infiltrating T cells in different cancer types, resulting in the construction of a scRNA-seq profile of T-cell composition in different cancer types. Cancer patients can be classified based on the T-cell composition, which is expected to provide new insights for precise immunotherapy targeting T cells. Wang et al. (2020) performed a comprehensive immune analysis of infants with biliary atresia (BA) by scRNA-seq, which showed that B-cell modification therapy alleviated liver pathology. Novel approaches linking antigen specificity to transcriptional dynamics by integrating T-cell receptor (TCR) tracking, scRNA-seq, TCR gene pools, and gene expression profiles from the same cells hold the promise of characterizing the specific clonality of immune cell subpopulations (De Simone et al., 2018).

Azizi et al. (2018) used scRNA-seq to analyze the immune cells of breast cancer patients, and found that the immune cells in the tumor microenvironmen (TME) have an increased “phenotypic volume” compared with immune cells in non-malignant breast tissues, indicating increased levels of phenotypic heterogeneity. In addition, another study conducted deep scRNA-seq on 12,346 T cells from 14 patients with NSCLC, describing the composition, evolution trajectory and functional status of tumor-infiltrating lymphocytes (TILs), results showed that based on CD8^+^ T cell phenotypes, cells which exhibited pre-existing state changes prior to exhausting had a better prognosis than those that exhausted directly (Guo et al., 2018). To investigate the molecular mechanism of B cell acute lymphoblastic leukemia (B-ALL) recurrence, Witkowski et al. (2020) demonstrated the remodeling of the B-ALL bone marrow immune microenvironment at disease onset, revealing the role of non-classical monocytes in B-ALL survival by SCS. Notably, Wilk et al. (2020) analyzed peripheral blood single nuclei cells from seven critically ill patients hospitalized for COVID-19 and constructed a cytogram of the peripheral immune response in these patients, which provided input for the invention of a new crown vaccine by SCS.

We have begun to gain a deeper understanding of immunotherapy, alongside the remarkable effects of anti-cytotoxic T lymphocyte-associated antigen-4 (CTLA-4) antibodies, anti-programmed death-1.

(PD-1)/programmed cell death-ligand 1(PD-L1) antibodies, and chimeric antigen receptor (CAR)-T cell therapies that include negative regulation of the body’s immune function in clinical cancer treatment. Schmidt et al. (2022) combined CRISPR screening and scRNA-seq to perform an in-depth molecular characterization of T cells in screening hits and uncovered the mechanisms regulating T-cell activation and the cellular states characterized by different cytokine expression profiles. This will likely inform the design of immunotherapies. In addition, a comprehensive understanding of the protein expression status of various types of TILs can greatly facilitate the design and development of next-generation tumor immune drugs. Lowery et al. (2022) localized 55 neoantigen-specific TCR clonotypes from ten metastatic human tumors to their single-cell transcriptome and identified CD8^+^ and CD4^+^ neoantigen-responsive TILs. Single-cell transcriptomics for immunotherapy studies allowed successful prediction of TCRs for cancer immunotherapy based solely on the TIL transcriptome status, opening a broader scope for the application of SCS technology. The transcriptomes of 17,000 cells from 18 primary or early recurrent hepatocellular carcinoma (HCC) cases were analyzed by Sun et al. (2021), and a decrease in regulatory T cells and an increase in dendritic and CD8^+^ T cells were observed in early relapsed HCC.

This provides more insight into the comprehensive description of the HCC ecosystem and directly reflects the significance and promise of SCS technology for innovative research in HCC: sc-RNA-seq reveals a unique immune ecosystem in early relapsed HCC. Intending to focus more on intratumoral immune heterogeneity, Zheng et al. (2020) obtained surface marker profiles of immune cells from T (HCC)/L (frontier)/N (non-tumor) samples from HCC patients and identified different L-region-specific immune cell profiles. Their data suggest a potential anti-tumor activity of double-positive T cells in HCC patients. Krieg et al. (2018) analyzed and compared immune cell subsets in the peripheral blood of melanoma patients before and after 12 weeks of anti-PD-1 immunotherapy using high-dimensional SCS and a bioinformatics pipeline. A significant response of T-cell blocks to immunotherapy was observed. Another study confirmed the presence of gene-expression signatures of three myoblastic cancer-associated fibroblasts (CAF) subpopulations at diagnosis is associated with resistance to anti-PD-1 antibiotics by single-cell analysis (Kieffer et al., 2020).

Cell therapy, represented by CAR-T cells, has made rapid developments in recent years, but there is still a lack of effective methods to analyze CAR-T cells after they enter the tumor microenvironment. Single-cell proteomics analysis cannot only obtain the activation status of CAR-T cells, but also determine the trend of CAR-T proteome changes at different time points, which can assist drug development scientists in the design and optimization of chimeric antigen receptors (CARs) (Bai et al., 2021). Xue et al. (2017) performed SCS of CD19 CAR-T pre-infusion products prepared from four healthy donors to assess the functional characteristics of CD19 CAR-T cells after antigen-specific stimulation. Single CAR-T cells exhibited significant cytokine secretion heterogeneity and a multifunctional (2+ cytokine) subpopulation that counteracted CAR bead stimulation. Therefore, multiple proteomic analyses revealed the diversity of responses of CD19 CAR-T cells to immune effectors, providing a new platform for obtaining detailed CAR-T cell data, and potentially providing an assessment of the safety and effectiveness of CAR-T cell therapy.

**FIGURE 5:**
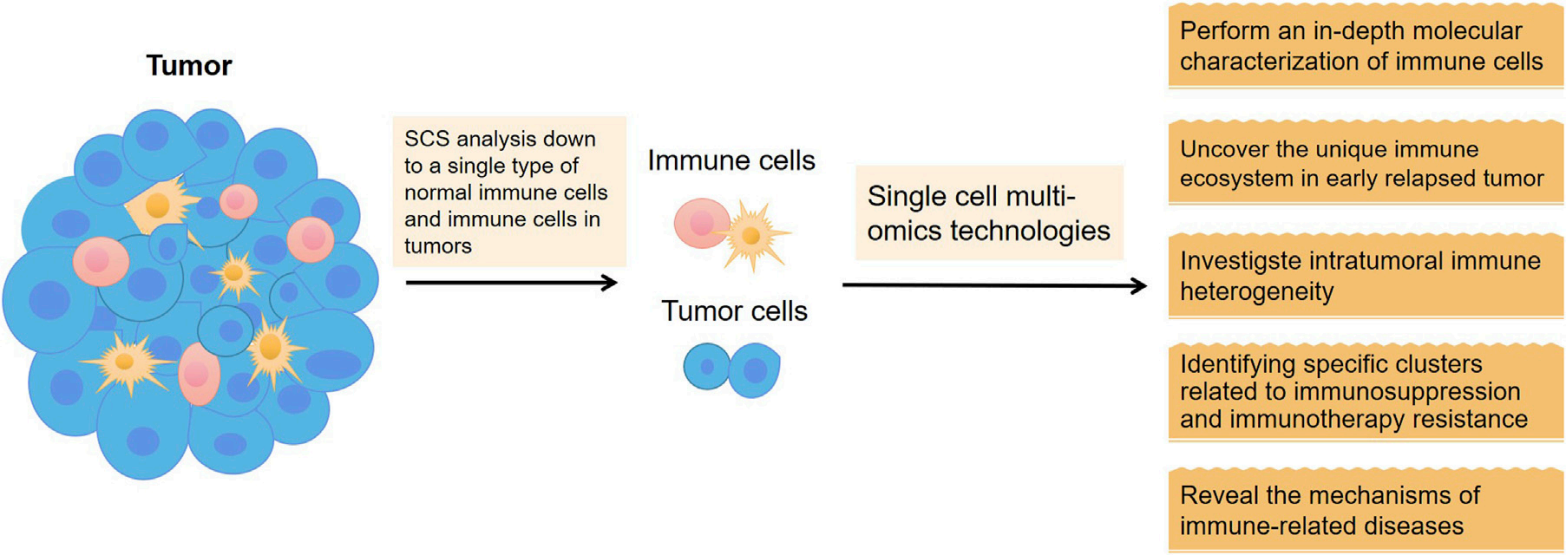
Application of SCS in tumor immunotherapy.

In summary, with the rapid development of molecular biology, molecular immunology, and related biotechnology, tumor immunotherapy has become a hot spot of clinical research. High-throughput, multifaceted characterization of the genomic, transcriptomic, and epigenomic features of tumors and relevant immune cells by SCS helps to dissect tumor heterogeneity and reveal complex interactions between tumor cells and their microenvironment (Gohil et al., 2021). Tumor immunotherapy research combined with SCS is expected to become an important comprehensive measure for tumor treatment in the future (Figure 5).

## 5 Conclusion

The completion of the draft human genome sequence was a key moment in genomics, heralding the advent of the “omics era”. Since the 1990s, human genomic research has contributed to the advancement of transcriptomics, proteomics, and metabolomics. SCS is gradually becoming an important tool for omics research.

This review systematically discusses the main classifications of SCS, isolation methods for single cells, the significance and challenges, and the excellent application potential of SCS. scDNA-seq atlases can provide both valuable data resources and biological insights that will facilitate cell engineering, regenerative medicine, and understanding of diseases. The introduction of an evolving lineage-tracing system with a scRNA-seq readout elucidates the hierarchical nature of tumor evolution and, more broadly, enables in-depth studies of tumor progression. scATAC-seq, an important breakthrough in single-cell epigenetics, reveals the accessibility of single-cell chromatin at the level of epigenomics, differentiates cell heterogeneity, and obtains information such as the location of open chromatin, binding sites of transcription factors, regulatory regions of nucleosomes, and the chromatin status. scCITE-seq obtains cell surface protein information and intracellular transcriptome information simultaneously, allowing for deeper differentiation of cell heterogeneity, more precise excavation of specific cell types, and exploration of the mechanisms behind biological phenomena including drug resistance. scSNARE-seq, which connects the transcriptome with the accessible chromatin of a cell, serves as a valuable tool for the input and output of transcriptional regulatory units to characterize tissue complexity and is highly useful for profiling cell maps of human tissues and clinical samples.

For CTCs, SCS is clinically important in pre-judgment, efficacy assessment, and monitoring of relapses and drug-resistant metastasis. In tumors, SCS provides a more intuitive platform to deeply explore the tumor microenvironment and heterogeneity, helping our understanding of how neoplastic tumors change their own surrounding microenvironment to escape host immune attack. SCS is expected to provide new therapeutic targets for anti-tumor drugs and promote the development of human medicine.

In conclusion, the application of single-cell omics sequencing technology is becoming more and more widespread and continues to grow in popularity. Based on its basic unit of research, the single cell, which is the foundation of all biomedicine, its future is immeasurable. The single-cell era has arrived, and SCS-based technologies will revolutionize biological science.

## Figures and Tables

**Table:** 

**APOBEC**	Apolipoprotein B mRNA editing enzyme catalytic polypeptide
**AR**	Androgen receptor
**B- ALL**	B cell acute lymphoblastic leukemia
**CAF**	Cancer-associated fibroblasts
**CAR**	Chimeric antigen receptor
**CEL-Seq**	Cell expression by linear amplification and sequencing
**CRISPR**	Clustered regularly interspaced short palindromic repeats
**CTCs**	Circulating tumor cells
**ctDNA**	Circulating tumor DNA
**CTLA-4**	Cytotoxic T lymphocyte-associated antigen-4
**CTM**	Circulating tumor microemboli
**EMT**	Epithelial mesenchymal transition
**ESCs**	Embryonic stem cells
**ESCC**	Esophageal squamous cell carcinoma
**FACS**	Fluorescence-activated cell sorting
**GC**	Gastric cancer
**hESCs**	Human embryonic stem cells
**ILCs**	Innate lymphoid cells
**IR**	Immune repertoire
**HCC**	Hepatocellular carcinoma
**LDCT**	Low-dose computed tomography
**LIF**	Leukemia inhibitory factor
**MDA**	Multiple displacement amplification
**MRD**	Microscopic residual disease
**NSCLC**	Non-small cell lung cancer
**PCA**	Principal component analysis
**PCR**	Polymerase chain reaction
**PD-1**	Programmed death-1
**PD-L1**	Programmed cell death-ligand 1
**revSCs**	Regenerative stem cells
**SCS**	Single-cell sequencing
**SCG**	Single-cell genomics
**scDNA-seq**	Single-cell DNA sequencing
**scRNA-seq**	Single-cell RNA sequencing
**scATAC-seq**	Single-cell assay for transposase accessible chromatin by sequencing
**scCITE-seq**	Single-cell cellular indexing of transcriptomes and epitopes by sequencing
**scMT-seq**	Single-cell methylation and transcriptome sequencing
**scSNARE-seq**	Single-cell single nucleus chromatin accessibility and mRNA expression sequencing
**scIR-seq**	Single-cell immune repertoire sequencing
**scCATCH**	Single-cell cluster-based annotation toolkit for cellular heterogeneity
**smart-Seq**	Switching mechanism at 5′ end of the RNA transcript sequencing
**SMART**	Switching mechanism at 5′ end of RNA template technology
**TAM**	Tumor-associated macrophages
**t-SNE**	T-distributed stochastic neighbor embedding
**TCR**	T-cell receptor
**TME**	Tumor microenvironment
**TILs**	Tumor-infiltrating lymphocytes
**UMAP**	Uniform manifest approximation and projection
**WGA**	Whole genome amplification
